# Cross-Cover Chaos to Calm: A Smarter Protocol for Efficient Patient Care

**DOI:** 10.1192/bjo.2025.10422

**Published:** 2025-06-20

**Authors:** Pinar Ozmen Akkurt, Martin Gracias, Seán Whyte

**Affiliations:** South West London and St George’s Mental Health NHS Trust, London, United Kingdom

## Abstract

**Aims:** To ensure fair workload distribution and faster patient care, SWLSTG follows a cross-cover policy, grouping wards into clusters based on proximity. Each ward has designated cross-cover doctors responsible for daytime medical advice and patient reviews in absence of the ward doctors.

A duty-doctor covers all the wards, handling emergencies, urgent reviews, and mandatory tasks if no cross-cover doctor is available. Duty-doctors carry the duty phone which is a dedicated mobile phone for urgent but non-life-threatening situations. However, in practice, ward staff often bypass the protocol and contact the duty-doctor directly, leading to:

Increased workload for a single doctor.

Delays in patient care due to unnecessary escalations.

Interruptions in emergency response.

This project aims to reinforce adherence to the cross-cover protocol, ensuring appropriate ward doctors are contacted first before escalating to the duty-doctor, reducing unnecessary workload and improving efficiency of patient care.

**Methods:** Pre-Intervention Data Collection: Distribute feedback forms to trainees to assess the frequency and impact of unnecessary duty-doctor calls.

New System Implementation: Set up an automatic voicemail on the duty phone using the Teams, reminding callers to contact their cross-cover doctor first (operating 9 am–5 pm on weekdays, except bank holidays). The Teams system also allows call tracking by displaying missed call numbers, unlike the previous system, which only showed “unknown number”.

Educational Intervention: Develop a leaflet and ward posters outlining the correct protocol, emphasising contacting cross-covering doctors first unless in a medical emergency.

Implementation: Circulate materials to nursing staff and ward teams, reinforcing adherence through staff meetings.

Collaboration with Ward Managers: Engage ward managers to reinforce adherence and ensure staff compliance.

Post-Intervention Evaluation: Conduct a follow-up survey to measure changes in behaviour and impact on patient care.

**Results:** The expected **Results:**

Faster response times for non-emergency reviews as cross-cover doctors are located closer to wards and responsible for fewer patients.

Improve efficiency of patient care by providing continuity.

Increase awareness among ward staff regarding the importance of cross-covering doctors.

Greater clarity and adherence to the protocol among staff.

Reduction in unnecessary escalation to duty-doctors.

**Conclusion:** Implementing a structured approach to protocol adherence improves workload distribution, reduces unnecessary escalations, and enhances efficiency of patient care. An automated voice message serves as a constant reminder, reinforcing the correct escalation process. However, Teams system carries potential downtime risk, so the old duty phone number will remain as a backup. Future steps include ongoing reinforcement, monitoring reliability, and periodic re-evaluation to sustain improvements.

